# Supplementary material for: Lineage tracing identifies heterogeneous hepatoblast contribution to cell lineages and postembryonic organ growth dynamics
Source: PLoS Biol. 2023 Oct 4;21(10):e3002315. doi: 10.1371/journal.pbio.3002315 (PMC10550115; doi:10.1371/journal.pbio.3002315)
Supplement: S1 Table — (DOCX) [file pbio.3002315.s002.docx]

**S1 Table.** Primer sequences:

| **Primer #** | **Sequence (5’ – 3’)** |
| --- | --- |
| 666 | GGAGCCATCTGATCGCAA |
| 667 | TAGGGATAACAGGGTAATCGAATT |
| 170 | CTGCAGGTCGGAGTACTGTC |
| 662 | TATATGTGGTCTTGATGTTTGC |
| 346 | GTTCAGGATCTCGATGCGGTG |
| 665 | CTGTATGTGGTCTTCAAGTTGCA |
| 663 | ACTGCTCCACCACGGTG |
