# Supplementary material for: Lineage tracing identifies heterogeneous hepatoblast contribution to cell lineages and postembryonic organ growth dynamics
Source: PLoS Biol. 2023 Oct 4;21(10):e3002315. doi: 10.1371/journal.pbio.3002315 (PMC10550115; doi:10.1371/journal.pbio.3002315)
Supplement: S1 Raw Images — (PDF) [file pbio.3002315.s003.pdf]

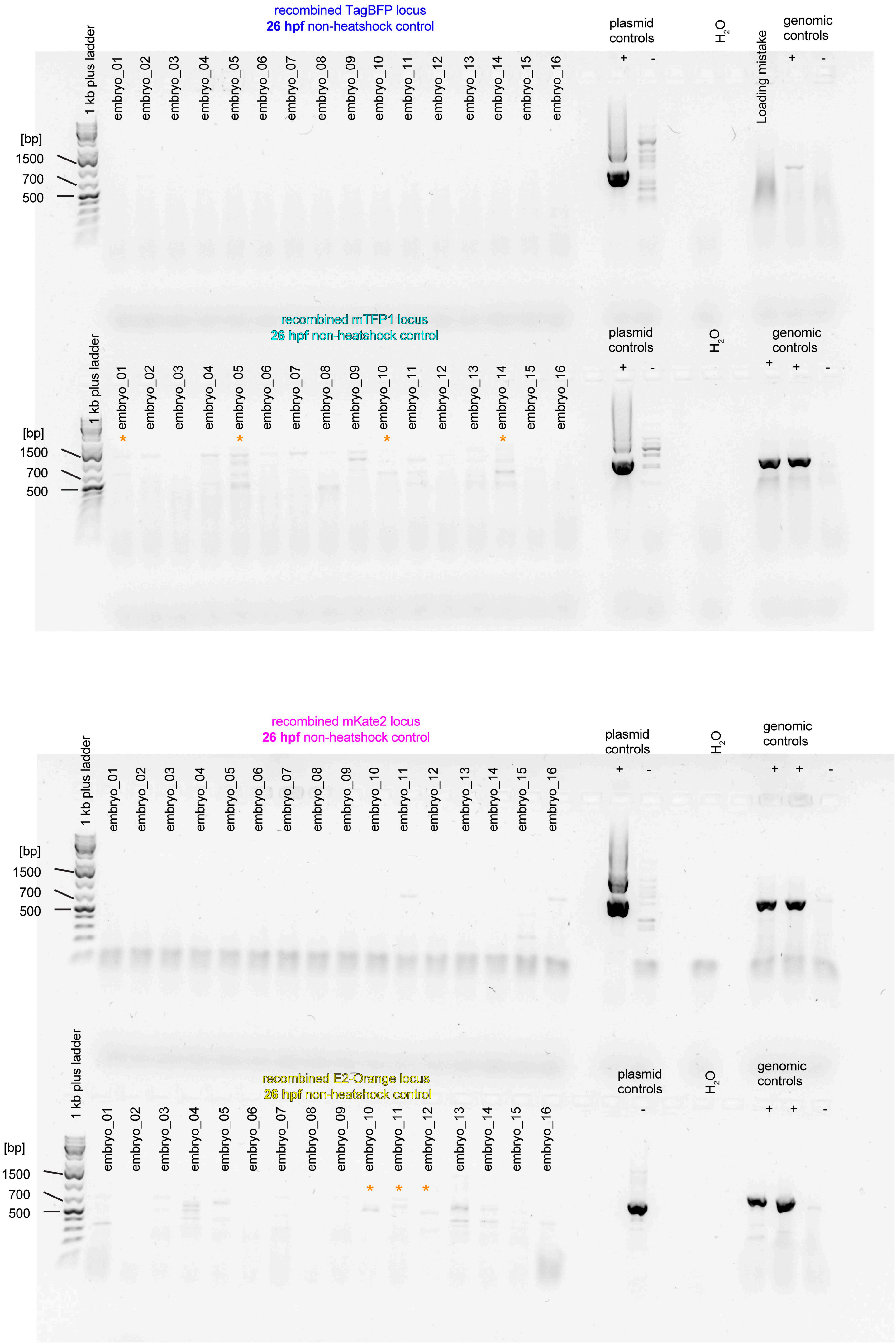
recombined TagBFP locus
26 hpf non-heatshock control
1 kb plus ladder
embryo_01
embryo_02
embryo_03
embryo_04
embryo_05
embryo_06
embryo_07
embryo_08
embryo_09
embryo_10
embryo_11
embryo_12
embryo_13
embryo_14
embryo_15
embryo_16
plasmid controls
+
-
H_2O
Loading mistake
genomic controls
+
-
[bp]
1500
700
500
recombined mTFP1 locus
26 hpf non-heatshock control
recombined mKate2 locus
26 hpf non-heatshock control
recombined E2-Orange locus
26 hpf non-heatshock control

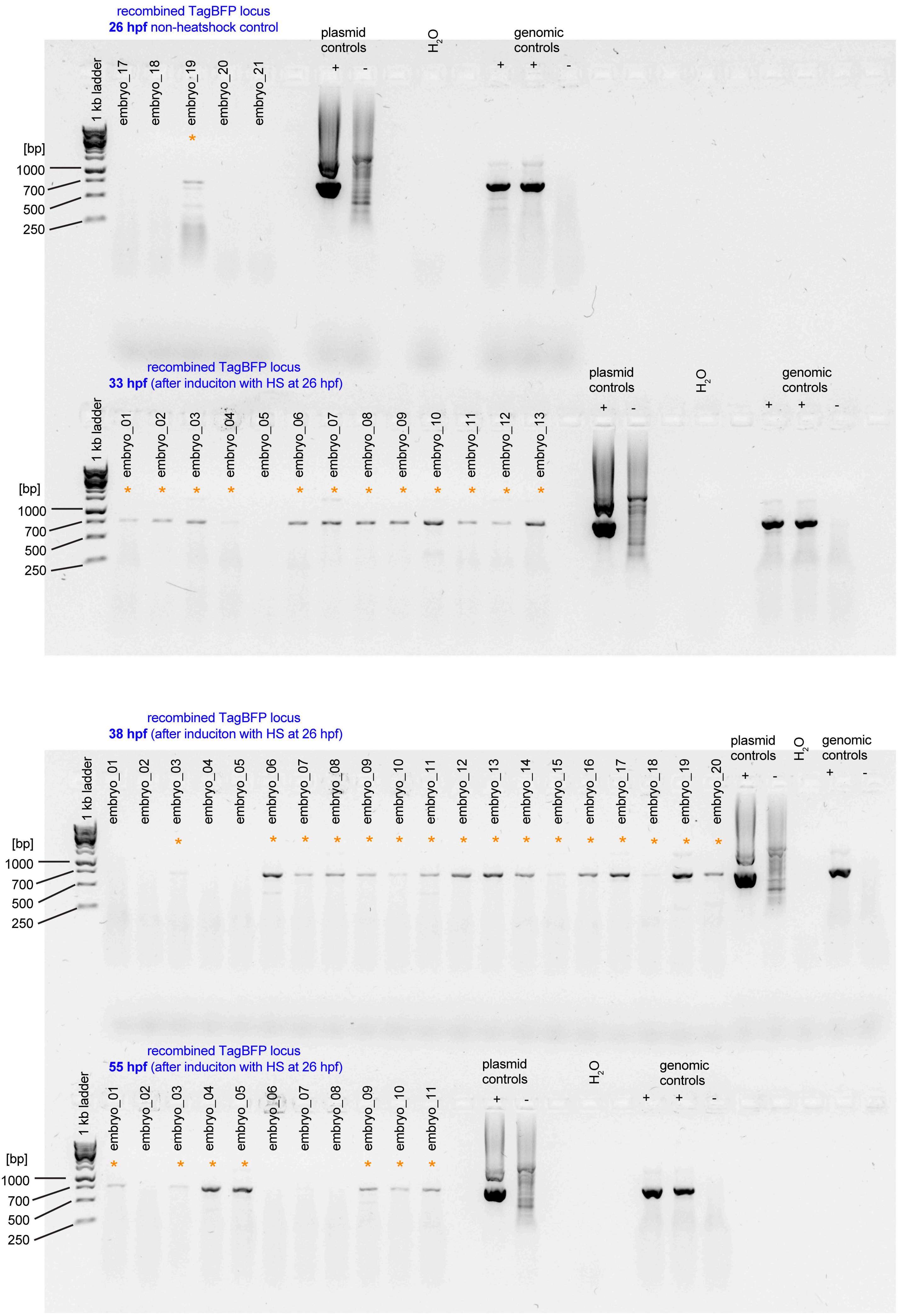

recombined TagBFP locus
26 hpf non-heatshock control
plasmid controls
H2O
genomic controls
1 kb ladder
embryo_17
embryo_18
embryo_19
embryo_20
embryo_21
+
-
+
+
-
[bp]
1000
700
500
250
recombined TagBFP locus
33 hpf (after induciton with HS at 26 hpf)
plasmid controls
H2O
genomic controls
1 kb ladder
embryo_01
embryo_02
embryo_03
embryo_04
embryo_05
embryo_06
embryo_07
embryo_08
embryo_09
embryo_10
embryo_11
embryo_12
embryo_13
[bp]
1000
700
500
250
recombined TagBFP locus
38 hpf (after induciton with HS at 26 hpf)
plasmid controls
H2O
genomic controls
1 kb ladder
embryo_01
embryo_02
embryo_03
embryo_04
embryo_05
embryo_06
embryo_07
embryo_08
embryo_09
embryo_10
embryo_11
embryo_12
embryo_13
embryo_14
embryo_15
embryo_16
embryo_17
embryo_18
embryo_19
embryo_20
[bp]
1000
700
500
250
recombined TagBFP locus
55 hpf (after induciton with HS at 26 hpf)
plasmid controls
H2O
genomic controls
1 kb ladder
embryo_01
embryo_02
embryo_03
embryo_04
embryo_05
embryo_06
embryo_07
embryo_08
embryo_09
embryo_10
embryo_11
[bp]
1000
700
500
250

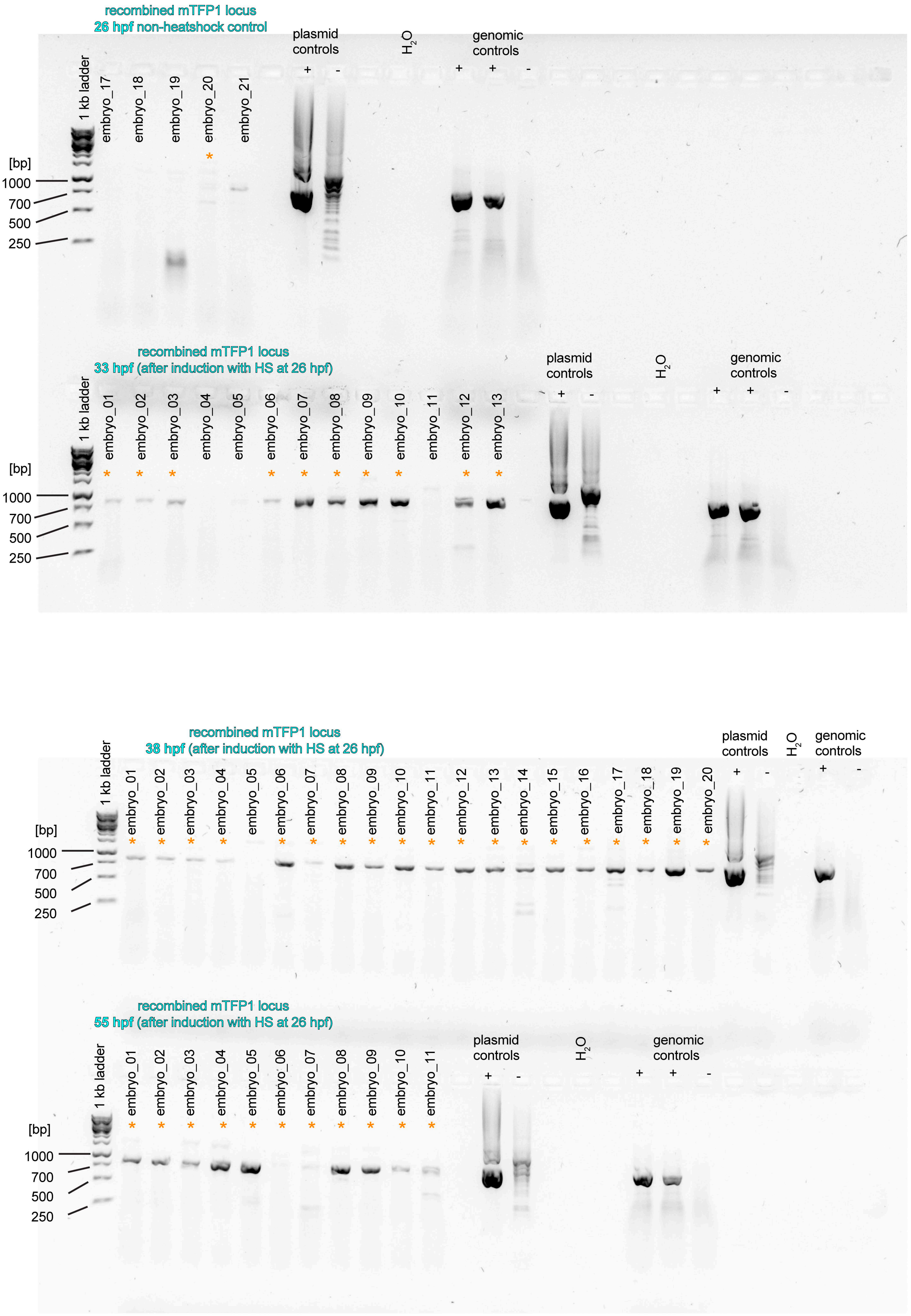
recombined mTFP1 locus
26 hpf non-heatshock control
1 kb ladder
embryo_17
embryo_18
embryo_19
embryo_20
embryo_21
plasmid controls
+
-
H_2O
genomic controls
+
+
-
[bp]
1000
700
500
250
recombined mTFP1 locus
33 hpf (after induction with HS at 26 hpf)
1 kb ladder
embryo_01
embryo_02
embryo_03
embryo_04
embryo_05
embryo_06
embryo_07
embryo_08
embryo_09
embryo_10
embryo_11
embryo_12
embryo_13
plasmid controls
+
-
H_2O
genomic controls
+
+
-
[bp]
1000
700
500
250
recombined mTFP1 locus
38 hpf (after induction with HS at 26 hpf)
1 kb ladder
embryo_01
embryo_02
embryo_03
embryo_04
embryo_05
embryo_06
embryo_07
embryo_08
embryo_09
embryo_10
embryo_11
embryo_12
embryo_13
embryo_14
embryo_15
embryo_16
embryo_17
embryo_18
embryo_19
embryo_20
plasmid controls
+
-
H_2O
genomic controls
+
-
[bp]
1000
700
500
250
recombined mTFP1 locus
55 hpf (after induction with HS at 26 hpf)
1 kb ladder
embryo_01
embryo_02
embryo_03
embryo_04
embryo_05
embryo_06
embryo_07
embryo_08
embryo_09
embryo_10
embryo_11
plasmid controls
+
-
H_2O
genomic controls
+
+
-
[bp]
1000
700
500
250

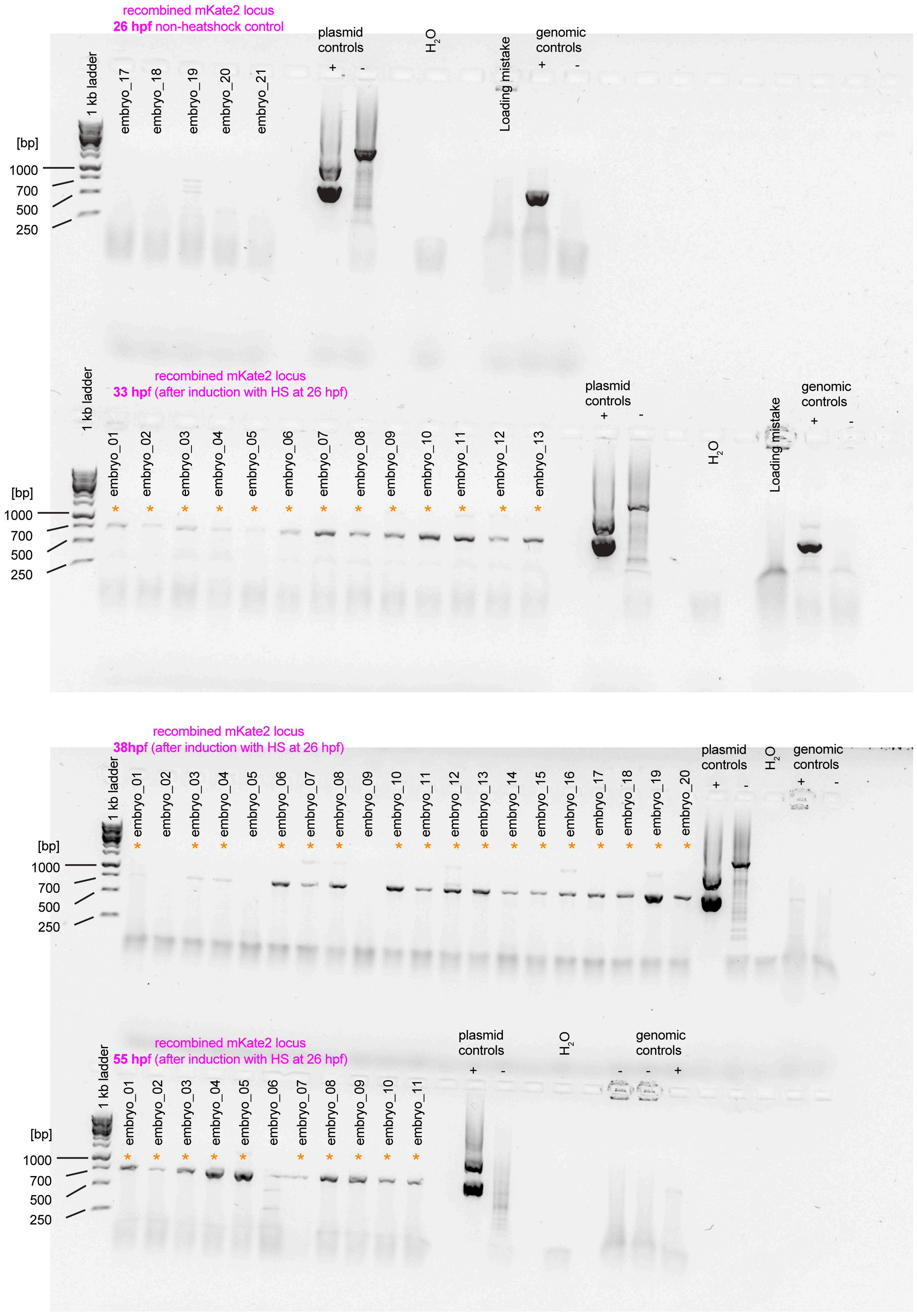

recombined mKate2 locus
26 hpf non-heatshock control
plasmid controls
H_2O
genomic controls
1 kb ladder
embryo_17
embryo_18
embryo_19
embryo_20
embryo_21
+
-
Loading mistake
+
-
[bp]
1000
700
500
250
recombined mKate2 locus
33 hpf (after induction with HS at 26 hpf)
1 kb ladder
plasmid controls
genomic controls
embryo_01
embryo_02
embryo_03
embryo_04
embryo_05
embryo_06
embryo_07
embryo_08
embryo_09
embryo_10
embryo_11
embryo_12
embryo_13
+
-
H_2O
Loading mistake
+
-
[bp]
1000
700
500
250
recombined mKate2 locus
38hpf (after induction with HS at 26 hpf)
plasmid controls
H_2O
genomic controls
1 kb ladder
embryo_01
embryo_02
embryo_03
embryo_04
embryo_05
embryo_06
embryo_07
embryo_08
embryo_09
embryo_10
embryo_11
embryo_12
embryo_13
embryo_14
embryo_15
embryo_16
embryo_17
embryo_18
embryo_19
embryo_20
+
-
+
-
[bp]
1000
700
500
250
recombined mKate2 locus
55 hpf (after induction with HS at 26 hpf)
plasmid controls
H_2O
genomic controls
1 kb ladder
embryo_01
embryo_02
embryo_03
embryo_04
embryo_05
embryo_06
embryo_07
embryo_08
embryo_09
embryo_10
embryo_11
+
-
-
-
+
[bp]
1000
700
500
250

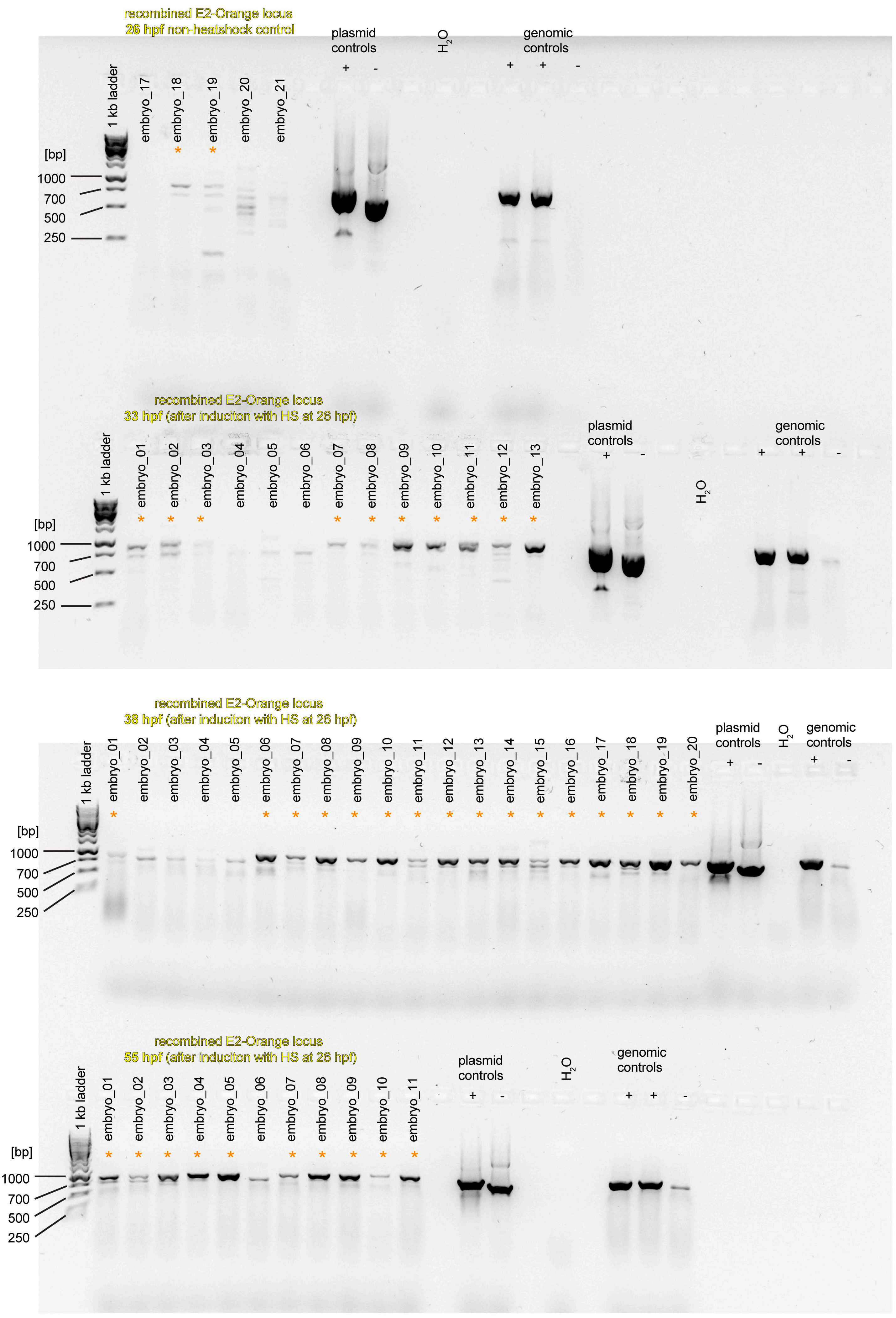
recombined E2-Orange locus
26 hpf non-heatshock control
plasmid controls
+ -
H2O
genomic controls
+ + -
1 kb ladder
embryo_17
embryo_18
embryo_19
embryo_20
embryo_21
[bp]
1000
700
500
250
recombined E2-Orange locus
33 hpf (after induciton with HS at 26 hpf)
1 kb ladder
embryo_01
embryo_02
embryo_03
embryo_04
embryo_05
embryo_06
embryo_07
embryo_08
embryo_09
embryo_10
embryo_11
embryo_12
embryo_13
plasmid controls
+ -
H2O
genomic controls
+ + -
[bp]
1000
700
500
250
recombined E2-Orange locus
38 hpf (after induciton with HS at 26 hpf)
1 kb ladder
embryo_01
embryo_02
embryo_03
embryo_04
embryo_05
embryo_06
embryo_07
embryo_08
embryo_09
embryo_10
embryo_11
embryo_12
embryo_13
embryo_14
embryo_15
embryo_16
embryo_17
embryo_18
embryo_19
embryo_20
plasmid controls
+ -
H2O
genomic controls
+ -
[bp]
1000
700
500
250
recombined E2-Orange locus
55 hpf (after induciton with HS at 26 hpf)
1 kb ladder
embryo_01
embryo_02
embryo_03
embryo_04
embryo_05
embryo_06
embryo_07
embryo_08
embryo_09
embryo_10
embryo_11
plasmid controls
+ -
H2O
genomic controls
+ + -
[bp]
1000
700
500
250
