# Supplementary figures and images for: Lineage tracing identifies heterogeneous hepatoblast contribution to cell lineages and postembryonic organ growth dynamics
Source: PLoS Biol. 2023 Oct 4;21(10):e3002315. doi: 10.1371/journal.pbio.3002315 (PMC10550115; doi:10.1371/journal.pbio.3002315)

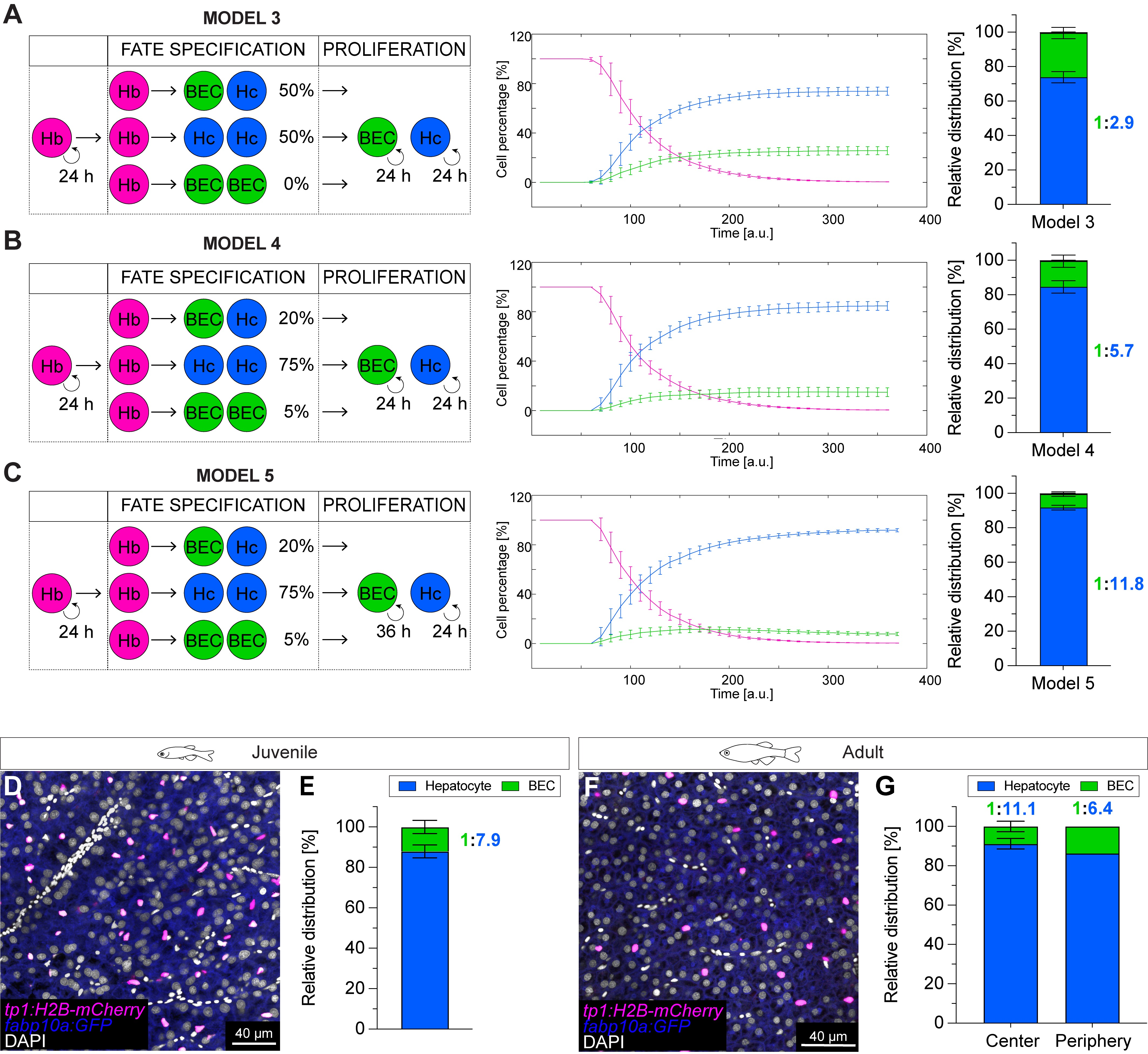

Supplement: S1 Fig — (A-C) Mathematical models simulating hepatoblast differentiation, based on heterogeneous hepatoblast potentials (A, B) or differential proliferation times (C; n = 10). (D, F) Presentation of 10 μm sections from juvenile (D) and adult (F) livers stained for fabp10a:GFP (hepatocytes), tp1:H2B-mCherry (BECs), and DAPI (nuclei). (E) Relative distribution of BECs and hepatocytes in juvenile liver (N = 4, n = 4 livers and 18 ROIs). (G) Relative distribution of BECs and hepatocytes at the organ centre (N = 1, n = 1 liver, 4 sections) or periphery in adult livers (N = 1, n = 1 whole-mount liver). The numerical values that were used to generate the graphs in (A-C, E, G) can be found in S1 Data. (TIF) [file pbio.3002315.s004.tif]

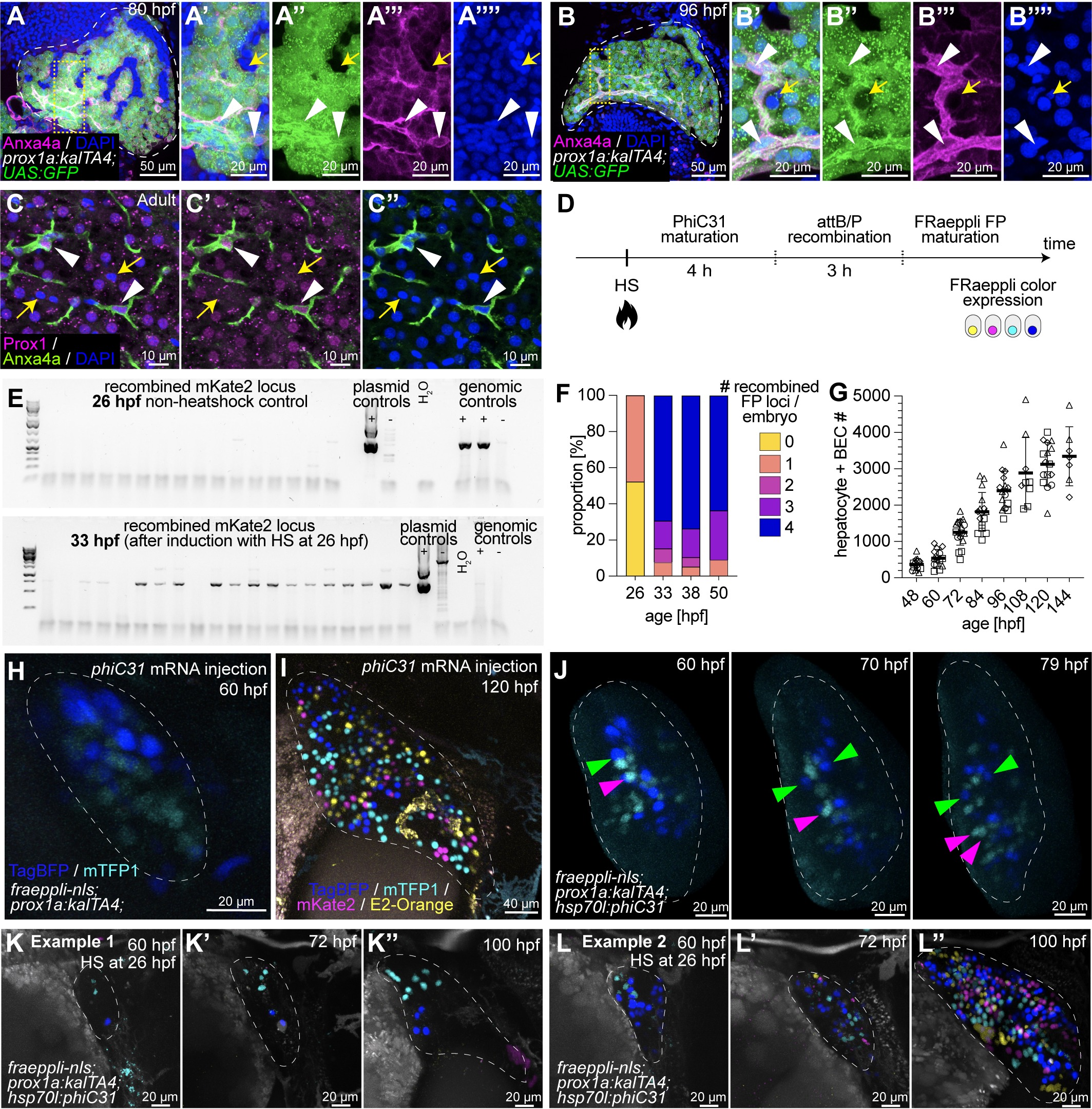

Supplement: S2 Fig — (A, B) (B) A 5 μm projection of tg(prox1a:kalTA4; UAS:GFP) embryos stained for 2F11 and DAPI at 80 hpf (A) and 96 hpf (B). White arrowheads indicate GFP+ BEC nuclei, and yellow arrows highlight GFP− endothelial cells. (N = 2, n = 8 livers). (C) A 10 μm projection of an adult liver section stained for Prox1 (magenta) and Anxa4 (green), white arrowheads indicate Prox1+ BEC nuclei, and yellow arrows highlight Prox1− endothelial cells. The Prox1 signal was filtered using a median filter with a 3-pixel kernel for better visualisation. (N = 2, n = 6 sections). (D) Schematic representation of the stepwise activation times of the fraeppli transgene. (E) PCR amplification of the mKate2 locus in individual embryos at 26 hpf or 33 hpf upon heat shock–mediated recombination at 26 hpf. (N = 2, n ≥ 16 embryos). (F) Distribution of the number of FRaeppli recombined loci per embryo upon heat shock at 26 hpf determined by PCR amplification of the recombined transgene. Band intensities at 26 hpf were about 4–6 times lower compared to later time points (N = 2, n ≥ 11 embryos). (G) Quantification of total liver cell numbers, encompassing hepatocytes and BECs, during development (N = 4, n ≥ 12 livers). Different shape data points indicate different experiments. (H, I) fraeppli-nls embryo activated by phiC31 mRNA injection showing only TagBFP and mTFP1 expression at 60 hpf (H), and expression of all 4 FRaeppli FPs at 120 hpf (n = 4 livers) (I). Temporal FP colour detection reflects the individual protein maturation times and depends on the strength of the respective Gal4-driver [32]. (J) Timelapse of TagBFP+ and mTFP1+ cells using spectral imaging of the liver upon heat shock induction at 9 hpf (N = 2, n = 3 livers). Some neighbouring cells stay close together (magenta arrow), while others move up to 20 μm apart (green arrows). (K, L) fraeppli-nls embryo reimaged at 60 hpf, 72 hpf, and 100 hpf. In sparse recombined embryos (K), not all 4 FRaeppli colours are expressed at 100 hpf and an i [file pbio.3002315.s005.tif]

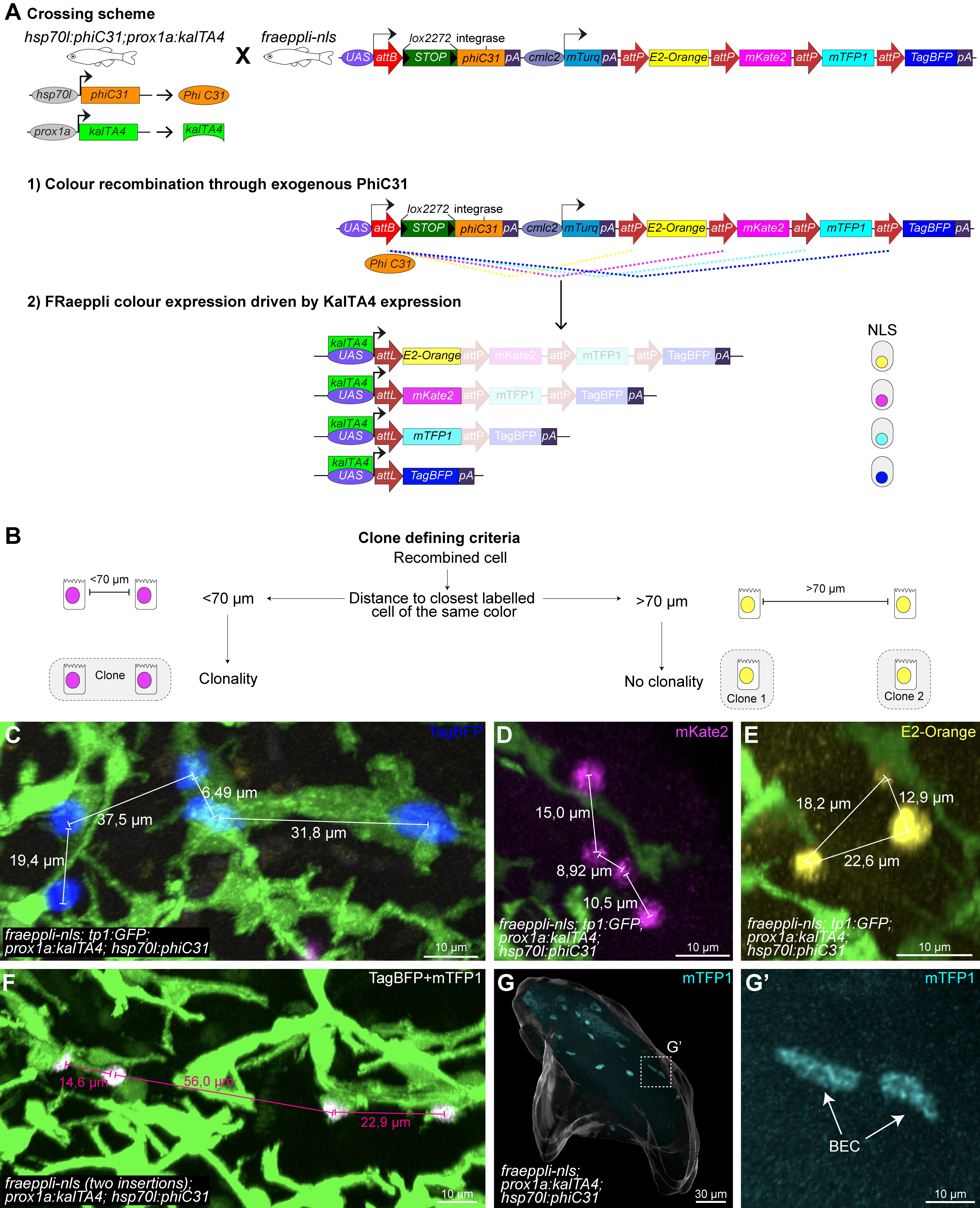

Supplement: S3 Fig — (A) Schematic representation of the crossing scheme of fraeppli-nls and hsp70l:phiC31; prox1a:kalTA4 fish. Exogenous PhiC31 integrase, expressed upon immediate temperature change of the medium, recombines attB/attP sites in the FRaeppli FP colour cassette. KalT4 drives the expression of the FRaeppli FPs. (B) Schematic showing the assignment criteria for manual clone definition based on distances between labelled cells of the same colour. (C-F) Intercellular distance within mixed clones (C), pure hepatocyte clones (D, E), or pure BEC clones (F); (C-F) correspond to examples shown in Fig 3D–3F. (G) Pure BEC clone, assignment based on the elongated cell shape. (TIF) [file pbio.3002315.s006.tif]

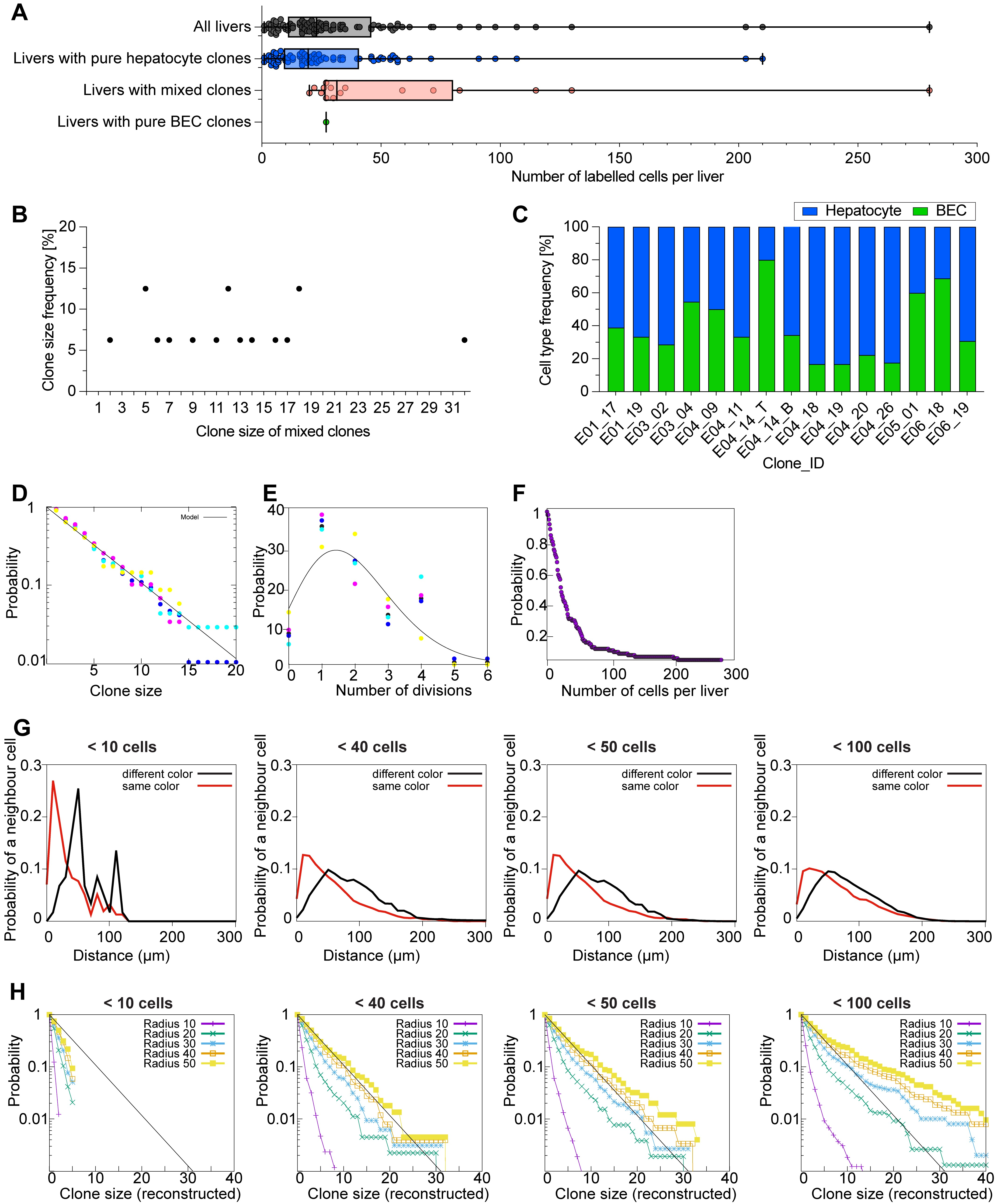

Supplement: S4 Fig — (A) Distribution of all labelled cells per livers. (B) Frequency of manually assigned mixed clone sizes (N = 5, n = 16 clones). (C) Cell type distribution within a mixed clone (N = 5, n = 16 clones). (D) Clone size distribution for different colours represented in a semi-log plot shows highly consistent values and good fit to a simple exponential distribution (black line), expected for a single population undergoing stochastic division. (E) Number of cell divisions of manually defined clones fit a Poisson distribution (black line) as expected for stochastic divisions. (F) Cumulative probability of a certain number of labelled cells per liver (N = 6, n = 97), showing that most livers have less than 50 labelled cells, but with heavy tails (10%–20%) of highly induced livers. (G) Probability that a given cell had a neighbouring cell with the same (red line) or a different colour (black line). Plots show subsets of the data that included livers with a total number of less than 10, 40, 50, or 100 labelled cells. Both distributions show high overlap for highly induced livers, which signifies poor clonality. For distances of less than 50 μm and livers with less than 40 cells, the ratio of “same” to “different” colour is high, meaning that nearby cells of the same colour are unlikely to be nonclonal. (H) Manually determined size distribution of clones (black line) plotted together with different reconstructed clone size distributions. Different lines correspond to the regrouping of neighbouring cells of the same colour in the same clone if present within defined radii. Plots show subsets of the data that included livers with a total number of less than 10, 40, 50, or 100 labelled cells. The numerical values that were used to generate the graphs can be found in S1 Data. (TIF) [file pbio.3002315.s007.tif]

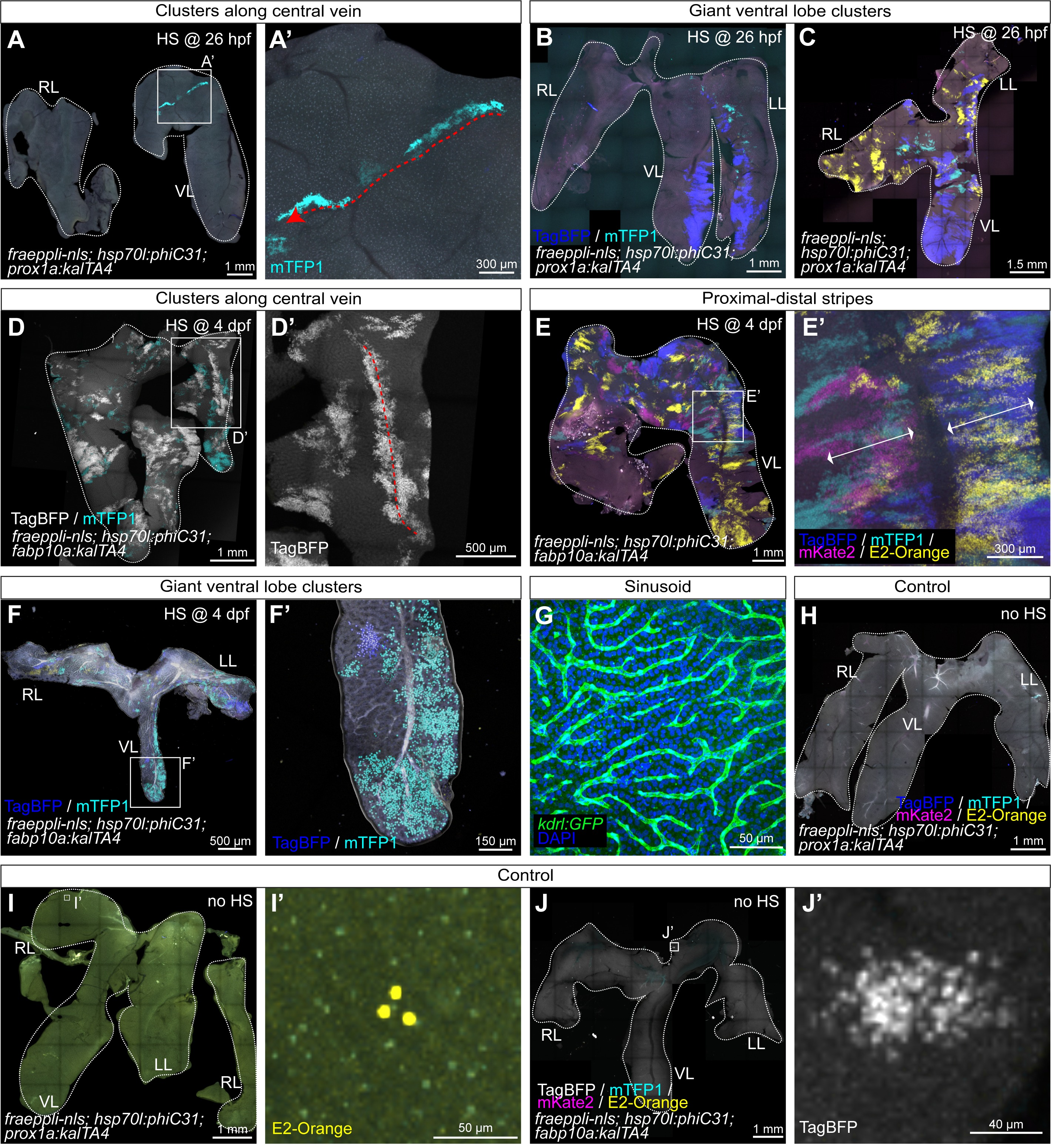

Supplement: S5 Fig — (A) Adult liver displaying clones along the central vein; recombination was induced in hepatoblasts at 26 hpf (n = 9 livers). (B, C) Adult livers exhibiting giant clusters in the ventral lobe (n = 3 livers). For (A-C) total numbers: N = 9, n = 79 livers. (D) Adult liver with a cluster along a central vein (n = 5 livers) and (E) clusters oriented in lateral stripes (n = 10 livers) upon recombination induced in hepatocytes. For (D, E) total numbers N = 4, n = 31 livers). (F) Giant clusters in the ventral lobe are also apparent in juvenile livers when labelling was induced at 4 dpf in hepatocytes (n = 1; total N = 5, n = 42 livers). (G) Confocal section showing the kdrl:GFP+ sinusoidal architecture in the adult liver counterstained with DAPI (N = 1, n = 2 livers). (H-J) No recombined cells were detected in 84% noninduced control livers of long-term lineage tracing experiments showed no recombined cells (H; N = 15, n = 84 livers), and the majority of recombined samples (N = 15, n = 100) show only one recombined clone of a few labelled cells (I, J; N = 15, n = 16 livers). (TIF) [file pbio.3002315.s008.tif]

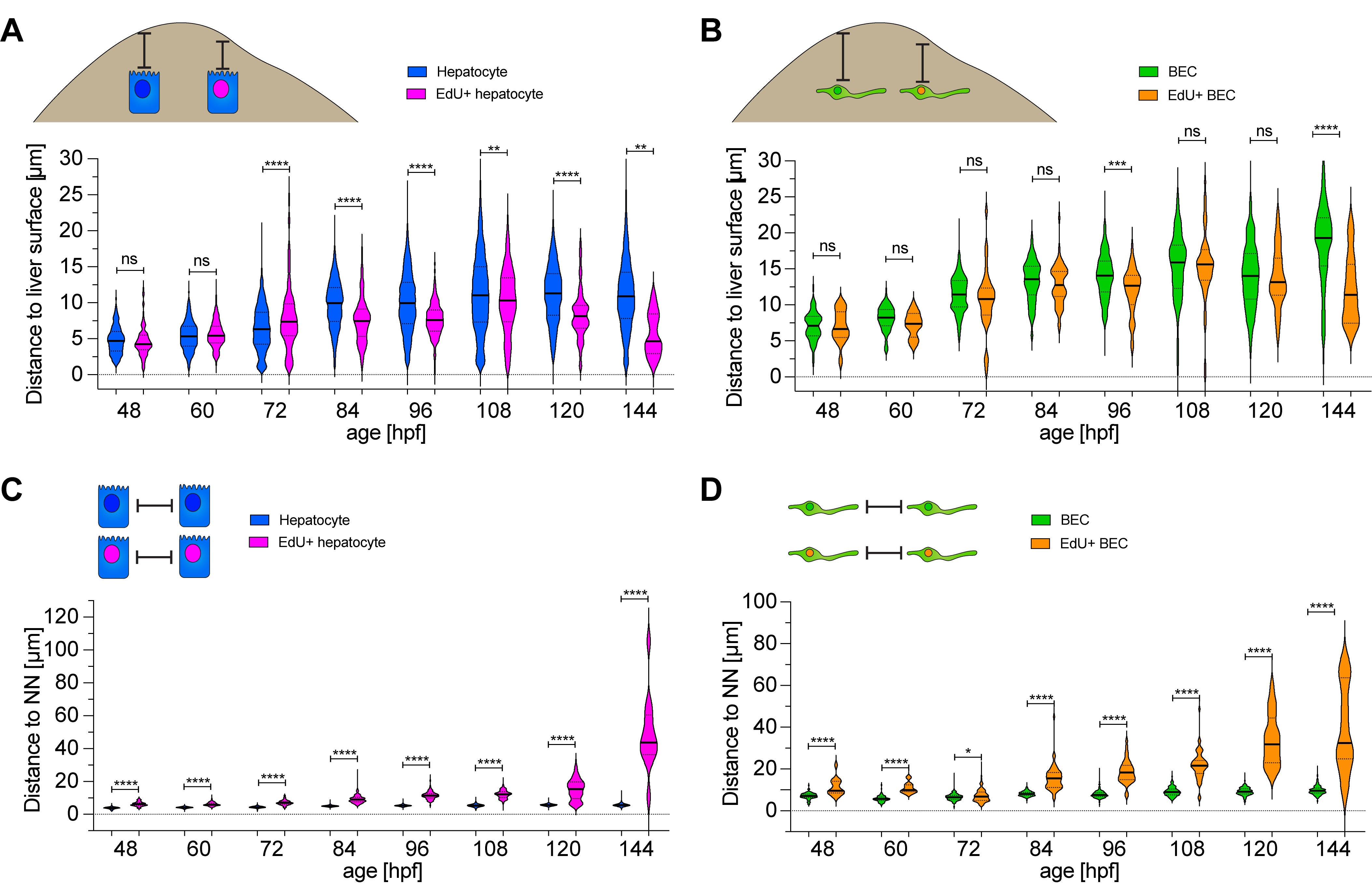

Supplement: S6 Fig — (A, B) Distribution of nuclear distance to the liver surface displayed for hepatocytes and EdU+ hepatocyte (A), and BECs and EdU+ BECs (B) (N = 2, n ≥ 6 livers). (C, D) Distribution of nuclear distance to the nearest neighbour (NN) shown for hepatocytes and EdU+ hepatocytes (C) and BECs and EdU+ BECs (D) (N = 2, n ≥ 8 livers). The numerical values that were used to generate the graphs can be found in S1 Data. (TIF) [file pbio.3002315.s009.tif]

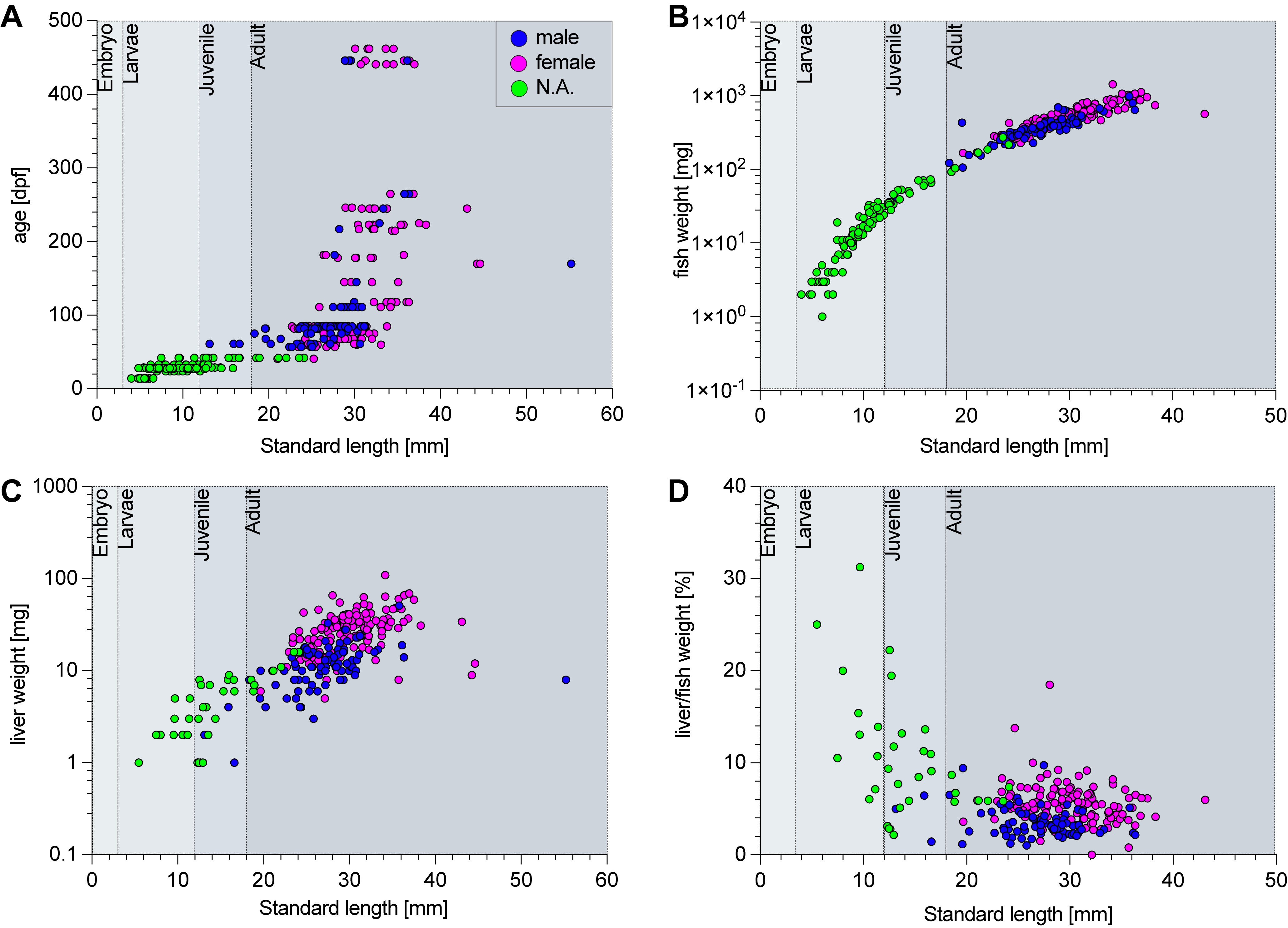

Supplement: S7 Fig — (A) Fish standard length (SL) plotted against fish age. (B, C) Fish weight (B) and liver weight (C) increases with SL represented in a semi-log plot. (D) Liver-to-body weight ratio during postembryonic growth is constant in adult fish. (N > 10, n ≥ 300 fish). Gender of the corresponding samples is colour coded: male (blue), female (pink), and ND (green). The numerical values that were used to generate the graphs can be found in S1 Data. (TIF) [file pbio.3002315.s010.tif]

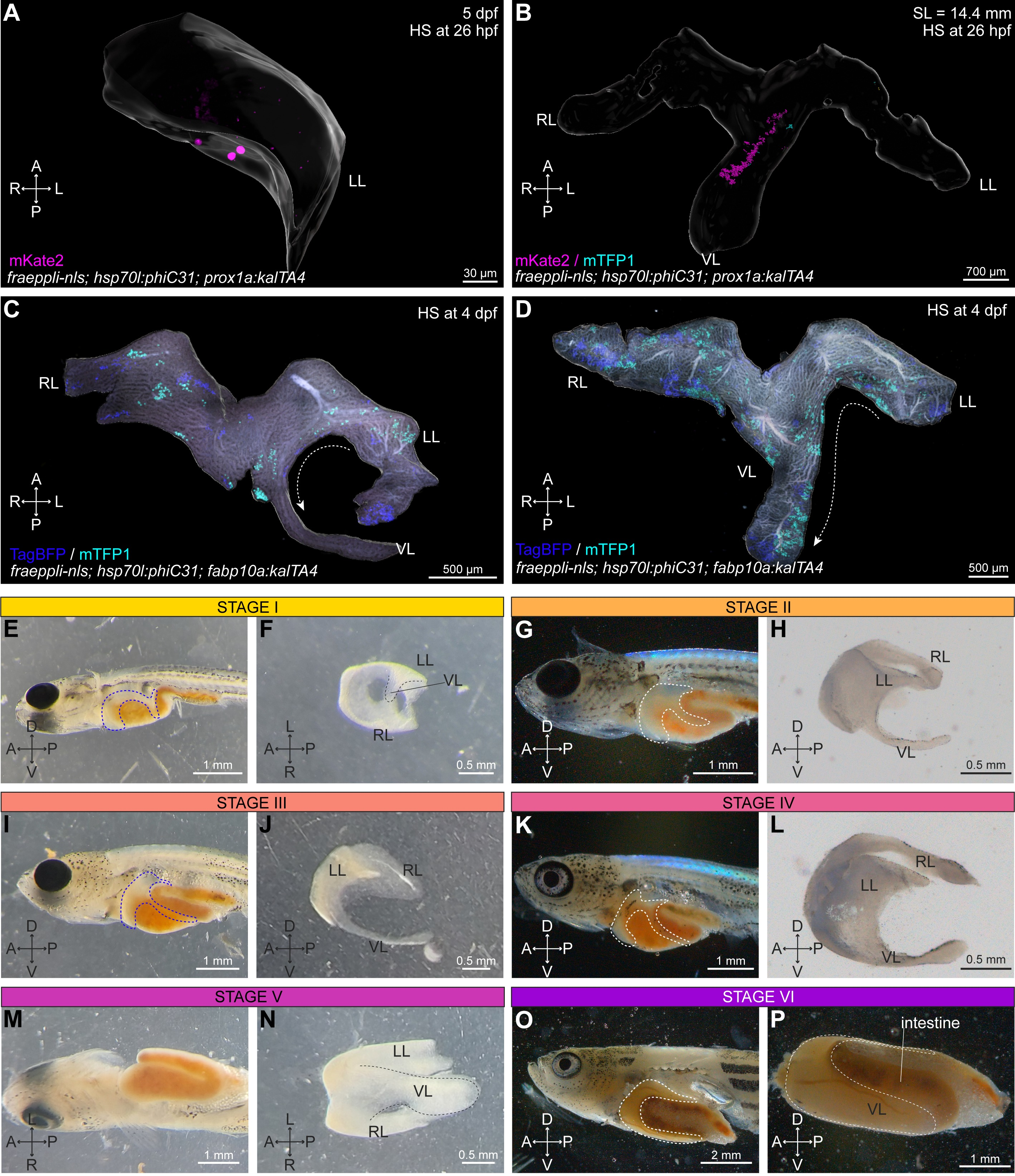

Supplement: S8 Fig — (A, B) Confocal images of the same liver showing the embryonic left liver lobe at 5 dpf with a 3-cell mKate2+ clone (A) and at juvenile stage (SL = 14.4 mm) including a continuous Kate2+ clone in the ventral lobe (N = 1, n = 1 liver). (C, D) Juvenile livers (C–SL = 8.46 mm and D–SL = 10.93 mm) with connected clusters that are oriented along the tissue edge and spread through the left and the ventral lobe. Arrows indicate cluster growth direction (N = 4, n = 14 livers). (E-P) Brightfield images of stages I-VI livers in loco within the fish (E, G, I, K, M, O) or dissected out (F, H, J, L, N, P). In (M), the liver is removed and the gut bend is visible. A, anterior; P, posterior; R, right; L, left; RL, right lobe; LL, left lobe; VL, ventral lobe. (TIF) [file pbio.3002315.s011.tif]
