# Supplementary material for: Lineage tracing identifies heterogeneous hepatoblast contribution to cell lineages and postembryonic organ growth dynamics
Source: PLoS Biol. 2023 Oct 4;21(10):e3002315. doi: 10.1371/journal.pbio.3002315 (PMC10550115; doi:10.1371/journal.pbio.3002315)
Supplement: Attachment — Submitted filename: PBIOLOGY-D-23-00163R1-response to reviewers_final.docx [file pbio.3002315.s012.docx]

**PBIOLOGY-D-23-00163R1 reponse to reviewers’ comments:**

We thank the reviewers for their positive response and constructive comments, which have helped us to improve our study. To address the raised points, we have made changes to the text and carried out more experiments and extended data analysis, including (i) genetic data corroborating the unbiased expression of the hepatoblast- and hepatic driver *prox1a:kalT4A* in both hepatocytes and BECs, (ii) an improved description of the multicolour lineage tracing system FRaeppli, including a detailed characterisation of the recombination and colour detection dynamics relevant to this study, and (iii) a quantification of the mixed BEC-hepatocyte clone size distribution. Moreover, we have modified the text especially the introduction and discussion focusing on the logic flow.

Since the submission of the original manuscript, no study directly relevant to our work has been published.

Please note, we exchanged panel Figure S4H, since we noticed while compiling the source data that we initially had added the results for 100 and 150 cells instead of 50 and 100 cells.

**Point-by-point response:**

**Reviewer #1:** This article by Unterweger et al studies the cellular origin and growth of the liver in the zebrafish. It combines mathematical models with genetic fate mapping and 3D organ imaging at single cell level followed by sophisticated statistical analysis to understand to which extent progenitor cells contribute to hepatocyte and biliary endothelial cell (BEC) generation.

If there are or not multipotent, bi- or uni- potent hepatocyte and BEC has been debated for very long. This clonal analysis using their recently published fraeppli-nls line combined with reporters and recombination lines specific for BECs and live progenitor cells respectively reveals new evidence on this question. The authors find that the liver is build from bi and unipotent progenitors. Their data suggests a ratio of 80% unipotent hepatocyte-producing hepatoblasts and 20% bipotent ones. The authors observe that clonal expansion associated to endothelial sinusoids and do not observe a clear pattern in the left and right lobes that would suggest that liver growth and shape is driven by directed proliferation. They also show that clonal expansion is probably stochastic, meaning that there is not a particular population with a higher degree of expansion. They do however observe that the ventral lobe has a different growth pattern with large clones found herein, what could indicate that few progenitors massively expands and give rise to this lobe. Further, they observe very unexpectedly,

that hepatocytes are polyploid transiently during growth, but in the adult polyploidy is lost.

The characterization of liver development absolutely exceptional. The author have performed an incredibly detailed and meticulous work, that will be of great use no only to understand cell fate determination of liver progenitors but also as an extraordinary helpful morphological atlas of zebrafish liver formation from the embryo to juveniles until adults.

Obviously, this nice work calls immediately for more questions:

How is the clone distribution in a pathological situation or injury scenario? What genetic programs make the cells decide to become a hepatocyte or a BEC? What happens to the polyploid hepatocytes?

However, these questions seem to go beyond the current article, that already counts with 8 main and 7 Supplementary Figures.

**Minor comment:**

* Making the data available might be useful for other researchers to analyse further parameters. Currently there is no statement on data availability.

We fully agree, data will be available in the supporting information, in line with the PLOS Data Policy.

* Page 10. Recombined liver clusters not livers

The sentence was indeed not clearly formulated. These results describe the percentage of recombined livers showing clusters with particular spatial topologies (e.g. stripe-like). This has been corrected; see line 271.

**Reviewer #2:** In this study, Ober and colleagues used mathematical modeling, quantitative biology, and multispectral lineage tracing to investigate the contribution of individual hepatoblasts to hepatic lineage formation and organ growth in the juvenile and adult fish. Such interdisciplinary approach unveiled many interesting features and novel mechanisms underlying liver organogenesis. First, they reported a constant ratio between the two main cell types in the liver, the hepatocytes and biliary epithelial cells, which is established at early embryonic stages and maintained throughout development, growth, and homeostasis in zebrafish. This ratio is the result of a heterogeneous lineage contribution of unipotent (the majority) and bipotent hepatoblasts to the liver, which is against the dogma of all hepatoblasts being bipotent. Then, by extending their lineage tracing analysis to adulthood, the authors discovered that embryonic cells contribute heterogeneously to postembryonic liver growth. Importantly, the findings reported in this manuscript suggest that both heterogenous lineage contribution and heterogenous proliferation dynamics during post-embryonic growth are not an intrinsic property of individual progenitors but more likely due to external inductive signals and surrounding microenvironmental niches (e.g., periphery, vessels, mesothelium).

Overall, I think this a strong paper and perfectly suited for PloS Biology. The results are compelling, and the methodology innovative and powerful. The manuscript is well-written and figures presented are of high quality.

The findings are extremely interesting, open up many new avenues of investigation in the field of liver biology (development & regeneration) and might have an impact on the development of future cell therapies for curing liver diseases, once conservation in mammalian species is assessed.

Minor points that **could be addressed in the discussion:**

- It would be important to discuss any contribution of cell death and/or competition to the heterogenous differentiation and expansion of progenitors during early liver development.

Indeed, we considered cell death as a contributing factor. However, based on our own work [Noel et al. 2008; Koltowska et al. 2013; Curado et al. 2010] and published work from other groups [e.g. Wu et al. 2017] there is commonly no cell death or very little during the relevant stages up to 5 dpf of liver formation. Cell death can be the result of cell competition, and while its absence indicates that cell competition likely plays no prominent role in the heterogenous expansion and differentiation of liver progenitors, we can formally not exclude it. This has been added to the text; see lines 411-413.

- The authors used the prox1 transgene as driver in the FRaeppli multicolour lineage tracing experiments. Can they exclude that the transgene labelling efficiency might be a confounding factor in the lineage tracing results?

This is a very good point. To show the labelling efficiency of the *tgBAC(prox1a:kalTA4)* line used to drive the expression of recombined *tg(fraeppli-nls)* loci in the liver, we crossed *tgBAC(prox1a:kalTA4)* with *tg(UAS:GFP)* carriers. These additional data show GFP expression throughout hepatocytes and BECs at 80 and 100 hpf, demonstrating that the *tgBAC(prox1a:kalTA4)* driver line shows no apparent spatial or cell type-specific bias. This corroborates its labelling efficiency for tissue-wide hepatoblast tracing and excludes confounding effects that might skew the results. These data are now included in Figure S2A-B” and the citation for the *tg(UAS:GFP)* line (Asakawa et al. 2008) has been added in the material and methods section.

Finally, we exchanged the panel showing Prox1 staining hepatocytes and BECs in adult liver sections with one including DAPI to underscore that all BECs and hepatocytes are positive; see FigS2C.

**Reviewer #3:** This is a review of MS entitled "Lineage tracing identifies heterogenous hepatoblast contribution to cell lineages and postembryonic organ growth dynamics" by Unterwegar et al. In this MS the authors use a newly developed (for zebrafish) lineage tracing mechanism termed FRaeppli-NLS. This system makes use of infrequent recombinasemediated activation of one of 4 fluorescent proteins to label and follow clones throughout development and into adulthood. Remarkably the non-overlapping spectra of the 4 FPs allow for reliable fate mapping and is even more powerful when combined with transgenic lineage markers and whole organ confocal analysis as performed herein. The authors aim to exploit this system, in combination with mathematical modeling, to understand hepatoblast potency during development and postnatally. Their models are produced by varying hepatoblast potency and proliferation in order to attain the 1:9 BEC :hepatoblast ratio observed at 120 hpf. BEC and hepatocytemarkers are used to experimentally determine that the 1:9 ratio is established at the onset of cell commitment @ 48 hpf. Edu labelling is used to identify proliferation rates and find a slight increase in BEC proliferation between 48-120 hpf, which is deemed too similar to hepatocytes to be significant. The FRaeppli system is used to assess hepatoblast potency by label clones 9-12 hours prior to commitment at 31-35 hpf, and imaging the resulting livers 100 hpf. Surprisingly they find that a minority of the hepatoblast clones are bipotential, while the majority gave rise solely to hepatocytes. Most of the clones produced fewer cells than expected based on their initial Edu experiments. They use a similar experimental strategy to label clones prior to specification and then allow their embryos to develop through juvenile and adult stages to examine how labeled clones contribute to the adult organ. They produce novel information demonstrating that hepatocytes clones develop

along central veins, or medial-lateral strives of hepatocytes and demonstrate, by imaging clones at 120 hpf and tracing over months, how the ventral lobe is formed. They find that most proliferation occurs from cells closest to the cell surface, demonstrating that the significant growth observed between 120 hpf through 3 mpf is spurred by dividing cells at the periphery of the organ and that polyploidy is not a mechanism utilized significantly by zebrafish during normal growth.

Overall this paper uses a novel and promising labelling system and the resultant confocal images are very detailed and aid in understanding how the liver is expands from progenitors. The most convincing data is that which describes how the liver develops post-embryonically. This data is mainly descriptive, and lacks a coherent theme, but is some of the first data produced from the zebrafish model. The embryonic data on hepatoblast potency is the most provocative and important however because this data is reliant on the reproducibility and defined occurrence of clone production there are several parameter gaps that must be sufficiently addressed. Furthermore, more attention should be paid to explaining the FRaeppli system, so that the data is accessible to a broader audience. Finally, introduction and discussion could be better focused as the logic in both is often difficult to follow.

Major weaknesses (in order of appearance in the MS, not of importance)

A) p. 3 last few sentences on the page. This part of the introduction is confusing. Ref 13: the authors methodically demonstrate that single cells are recombined at E8.25, coincident with hepatic specification. Demonstrating that the earliest hepatoblasts are bipotent. Ref12: demonstrates some E10.0 hepatoblasts are unipotent while most are bipotent. Couldn't this be seen as a gradual restriction of hepatoblast fate over time? The end of this paragraph is not focused and warrants more attention.

This is an important point, and we agree that both studies referred to in the introduction suggest that the fate of endodermal foregut progenitors is gradually restricted, first to bi-potent hepatoblasts and subsequently to uni- and bipotent hepatoblasts before they finally only give rise to hepatocytes (ref 12+13; now ref.9+10). Both studies are important in the field, yet ref. 13 (now ref 10) labelled foregut progenitors and therefore too early, and ref.12 (now ref.9) used the Lgr5-driver labelling a subpopulation of hepatoblasts and importantly only analysed a subset (2%) of labelled cells restricted to the portal vein area. Therefore, an organ-wide understanding of lineage decisions underlying liver formation and the establishment of cell type proportions is still missing. The text was changed to improve the focus of the section; see lines 66-83.

B) Fig 1B' Are the grey cells the HNF4a + cells? What is the difference between the grey cells and those with a white core?

An excellent point, the bright cells are blood cells exhibiting high autofluorescence upon 488nm excitation, which were therefore detected together with the Hnf4a signal in this experiment. The higher fluorescence signal together with their ellipsoid shape allows to distinguish blood cells from hepatocytes, exhibiting a lower signal and round shape, and thereby exclusion from this proliferation analysis. This is now described in the figure legends; see line 516.

C) Fig 2: An image used for cell proliferation should be included in Figure 2.

A representative image of an EdU staining showing both proliferating hepatocytes and BECs at 72 hpf has now been included in Figure 2.

D) Fig 2: What do the shapes in each category represent? How many livers were examined at each stage? Why are there fewer shapes at 144hpf?

The different shapes represent independent experiments. For all stages we analysed a minimum of 8 embryos, which were derived from 3 independent experiments for all time points between 48-120 hpf, and from 2 independent experiments for 144 hpf. Therefore, there are 2 instead of 3 different shapes represented in the 144 hpf data set. This information is provided in the accompanying figure legends, see line 535.

E) Fig 2: It appears as if the trend is that BECs differentiate faster at all time points (with significance at 96 and 120hpf). This point is not mentioned in the text other than "minimal differences between BECs and hepatocyte proliferation rates" are observed. Please describe how such changes might affect the model and if others have observed such differences in proliferation rates.

The reviewer refers to the finding that a greater proportion of BECs than hepatocytes show EdU incorporation at 96 and 120 hpf. EdU labels cycling cells in S phase however does not provide any information concerning proliferation times of either cell type.

Assuming a fraction of BECs would exhibit a faster doubling time/cell cycle than hepatocytes, this would shift the BEC:hepatocyte ratio in a model towards BECs.

To our knowledge, no systematic analysis comparing BEC and hepatocyte proliferation covering all stages of liver development between 48-144 hpf has been published. Solely, individual fish [Lorent et al. 2010] have been analysed.

F) p. 8: A more complete description of the Fraeppli-NLS system is needed for the reader to critically evaluate this experimental system. For example- what are the dynamics of the detection? How soon after recombination can the clones be visualized? How many cells are in each clone at the onset of detection? Most importantly- how long does it take for all clones to be established? Do the number of clones increase after differentiation at 48 hours? If so, then could the small number of cells in a clone reflect a developmentally later onset of recombination? This could explain the higher number of smaller than expected clones.

For a more detailed description of the FRaeppli-NLS system and to address the points raised concerning the dynamics of fluorescent protein and clone appearance/detection, we performed more experiments:

(i) By live-imaging, robust hepatic BFP and TFP expression was detected at 60 hpf after heat-shock induction of phiC31 at 26 hpf. In sparsely recombined livers, individual clones can be defined at 60 hpf, at the onset of detection, revealing mostly 1- or 4-cell clones (new Figure S2K), while in highly recombined livers labelling is too dense and thereby prevents definition of individual clones and their cell numbers (new Figure S2L).

Successive imaging of the same embryos at 72 and 100 hpf, revealed additional clones expressing mOrange or mKate2 (new S2L’,L’’), reflecting individual protein maturation times and in line with previous results (Caviglia et al 2022). The temporal lag between recombination and colour detection is also influenced by the strength of the respective Gal4/KalTA4 driver, e.g. FRaeppli colours are detected 24 hours after heat-shock when using the hepatocyte driver *fabp10a:kalTA4*.

(ii) To monitor the temporal dynamics of *fraeppli-nls* recombination in embryos, we designed PCR experiments to detect the recombined genomic loci of the individual fluorescent proteins at multiple timepoints after heat-shock-mediated induction of phiC31 expression. Already at 33 hpf, seven hours after heat-shock-mediated induction of phiC31 expression at 26 hpf, all embryos exhibit successful recombination and most embryos of all four fluorescent proteins (new Fig. S2E,F). These temporal dynamics of recombination mediated by exogenous phiC31 are similar to those in the early embryo (Caviglia et al. 2022). In summary, these results show that recombination is detectable at 33 hpf in hepatoblasts.

Moreover, the fraction of embryos displaying recombination of all four fluorescent protein loci remained constant at 38 and 50 hpf, indicating immediate and efficient recombination upon phiC31 maturation; these results are depicted in Figure S2D-F. Yet, we can formally not exclude unspecific recombination events occurring later during development that may contribute to the pool of small clones. This statement has therefore been added to the text; see 250-253.

G) p.8 In the 2022 Caviglia et al paper defining this method, Cre is used to induce recombination of the fluorescent reporter. It is not clear to this reviewer how Cre is induced in the system used in the current manuscript.

Thank you for highlighting this important point. There are two different strategies to induce the recombination of the FRaeppli-NLS cassette: (i) Cre-based recombination removes a STOP codon initiating expression of the inbuilt phiC31 integrase, which in turn mediates attB-attP recombination resulting in fluorescent protein expression. (ii) an alternative 1-step activation strategy independent of Cre utilizes exogenous phiC31 integrase for direct attB-attP recombination. Both strategies are described in Caviglia et al. 2022.

In this manuscript, we use the latter strategy for better temporal control. Exogenous phiC31 integrase is provided by the transgenic *hsp70l:phiC31* line, which expresses *phiC31* under the control of the *heat-shock 70 kd protein-like (hsp70l)* promoter, which is activated by an abrupt shift in medium temperature. This has been addressed in the text, see lines 185-188 and in agreement with the reviewer’s point, a new schematic of the stepwise process has been added as Figure S3A.

H) Fig 3D: This image is not convincing and appears to be two different clones, each clone labeled with the same color, one differentiated toward hepatocyte the other toward BEC. It is also striking that there are similarly large numbers of cells in each lineage. In this example the hepatocytes and BECs are in discreet positions of the liver and each lineage is clustered together. Do all bipotent cells display a similar spatial organization (in distinct portions of the liver)?

We agree that the initially shown panel was not the clearest example. We now replaced panel 3D with a more representative example. In fact, hepatocytes and BECs of one single clone, as determined by the clone-defining ruleset, seldomly display such a discreet spatial organisation. In most cases hepatocytes and BECs of one clone are intermingled and not separated from each other and they can be found in any position within the liver.

I) Fig3: Is there a difference in the number of cells in clones that display a unipotent hepatoblast fate and those that display a bipotent hepatoblast/BEC fate?

To address this point, we have quantified the overall cell number of mixed hepatocyte-BEC clones and now integrated in Figure S4, see panel S4B and C. The size range of hepatocyte-BEC clones is within the range of the previously determined hepatocyte clones shown in Figure 4A. Moreover, hepatocytes and BECs are represented in varying proportions within a given clone; see Figure S4C).

J) Fig 3. The distribution of clone types (represented by D,E,F) within the 85 labelled livers should be provided. For example- this could demonstrate that hepatoblast-only clones are found throughout most of the labeled livers or are restricted mainly to those livers that do not have bipotent clones. Given that the number of cells produced by any one clone typically did not undergo as many cell divisions as predicted, is it possible that the cells were labeled later than expected?

This is indeed an important point. Due to the fewer number of mixed BEC-hepatocyte clones, the majority of livers contained only FRaeppli-labelled hepatocytes. In 20 livers, mixed clones were always detected alongside pure hepatocyte clones. Of these, the majority showed one mixed clone, and three livers contained two mixed clones. This finding indicates that the more rarely detected mixed clones are comparatively evenly distributed among the labelled livers.

Given the labelling variability between individual livers, we were not able to assign every labelled cell to a clone, in particularly in more densely recombined livers. This unfortunately prevents to describe the exact distribution of clone types per liver. Yet, by quantifying the number of all labelled cells per analysed liver revealed that the detection of mixed BEC-hepatocyte clones increased with greater labelling density, suggesting distribution of clone types correlated with labelling density; these data are now shown in Figure S4A.

Our PCR results characterising *fraeppli-nls* recombination dynamics show that recombination occurs fast (Figure S2E,F), revealing that within seven hours after PhiC31 integrase induction almost all embryos recombined all four FRaeppli fluorescent protein loci. This strongly suggests that *fraeppli-nls* recombination and thereby hepatoblast labelling occurs within a short time window after the heat-shock, although we cannot exclude the possibility of occasional unspecific recombination. Overall, this is supported by rare and low level unspecific FRaeppli fluorescent protein expression observed in 1.8% of control embryos (see lines 250-252) without heat-shock.

K) Page 9: paragraph 2. The reason that the hepatocyte clones were focused on rather than BEC clones is confusing.

Our analysis initially focused on hepatocyte clones since the distinct elongated BEC morphology and their network organisation challenged defining BEC-containing clones. To overcome this, we performed additional experiments using two *fraeppli-nls* insertions generating unique colour combinations corroborating that the same ruleset for defining hepatocyte clones applies to BEC-containing clones. Hence, individual BEC nuclei of the same colour not exceeding a maximal distance of 70 μm are assigned to the same clone; this has been incorporated in the text see 222-223 and Figure S3B-F, S4B,C. For consistency of the figure we exchanged the originally pure BEC clone lacking the transgenic *tp1:GFP* expression with one from the data set using 2 insertions and moved the original example to Figure S3G.

Minor Edits

A) Fig 3B: Why does the timeline extend to 120+ hours? The paper states that embryos are fixed and imaged at 100 hpf. Furthermore this timeline is confusing when contrasted with Fig 5 that states time of imaging and time of fixation.

The timeline in Figure 3B extended to >120 hpf solely because we used 24-hour units. Moreover, the information indicating fixation and imaging timepoints has been unified.

Both points have been implemented, see Figures 3B,C and 5B.

B) Fig3. The legend for D-F states that N=6. What does the N represent?

For experiments shown in D-F, N=number of individual experiments and n=number of clones; this information has been systematically updated in all relevant figure legends (see also next point).

C) Figure 5: How many total livers were examined? What do the N and n represent in this figure legend?

For this set of experiments, N=number of individual experiments and n=number of livers; the figure legend has been updated accordingly, see also previous point of the reviewer.

D) Discussion: page 16. The second full sentence is not complete.

We agree and have modified the original sentence as shown below; now lines 422-424.

Original: *This is supported by early cholangiocyte committed hepatoblasts, so far only been deduced computationally from single-cell RNA sequencing of murine hepatoblasts at E10.5 [12].*

New: *Our result thereby provides lineage-tracing evidence for the presence of early BEC-committed hepatoblasts, which have so far only been proposed based on single-cell RNA sequencing of murine hepatoblasts at E10.5 [9].*

E) Page 17: The sentence beginning "the overproportionate contribution…" is difficult to comprehend.

Thank you for pointing this out. We rephrased it as shown below; see lines: 462-465.

Original: *The overproportionate contribution of these giant clusters to the adult organ, therefore, likely reflects that only a few embryonic cells compared to the two dorsal lobes produce a lobe of similar size and is reminiscent of the clonal dominance observed in other tissues [27,61,62].*

New: *Given all three lobes are of similar size in the adult liver, the overproportionate contribution of these giant clusters to the mature ventral lobe, therefore suggests its origin from far fewer embryonic cells than the two dorsal lobes, which is reminiscent of the clonal dominance observed in other tissues [27,61,62].*

F) Page 17: Final paragraph " We propose that both lineage and proliferation heterogeneity is not intrinsic hepatoblast property…" Insert "an" before "intrinsic".

The text has been adjusted as suggested; see line 482.
