# Supplementary material for: Lineage tracing identifies heterogeneous hepatoblast contribution to cell lineages and postembryonic organ growth dynamics
Source: PLoS Biol. 2023 Oct 4;21(10):e3002315. doi: 10.1371/journal.pbio.3002315 (PMC10550115; doi:10.1371/journal.pbio.3002315)
Supplement: Attachment — Submitted filename: PBIOLOGY-D-23-00163R2_response to reviewers_final.docx [file pbio.3002315.s013.docx]

We thank the reviewers for their positive feedback and additional careful and constructive suggestions for the manuscript, which have really helped us to further improve the manuscript. The revised manuscript now includes more precise descriptions and conclusions, in particular focusing on transiently higher BEC doubling times and the temporal dynamics of PhiC31 expression.

**Reviewer #2:** In the revised version of this manuscript the authors have satisfactorily responded to all the points raised by this referee.

The text revision and new set of experiments included has greatly improved the work.

We thank the reviewer for the positive feedback.

**Reviewer #3,** Kimberly D Tremblay (note, reviewer 3 has signed this review): PBIOLOGY-D-23-00163 Review #2

I appreciate the authors detailed response to many of my previous critiques. Several addressable yet important concerns were not answered satisfactorily.

We very much appreciate the constructive feedback from the reviewer, which allowed us to be more precise.

1)      The response to query E regarding Figure 2:

Figure 2E clearly demonstrates that BECs exhibit significantly higher proliferation rate than hepatocytes at 96 and 120 hours.  Line 154 states "our results reveal minimal differences between BEC and hepatocyte proliferation rates suggesting progenitor potential as a major factor for the establishment of the 1:9 BEC to hepatocyte ratio in vivo..".   Please explain how this proliferation data reveals "minimal differences in proliferation rates".  If as explained in your rebuttal that Edu does not represent proliferation rate, then explicitly remark on how the data in figure 2 supports minimal differences between BEC and hepatocyte proliferation rates.  If these results are incongruous with the proposed models explain how.

Revisiting the temporal differences in EdU incorporation in BECs , we have adjusted the text and removed the ‘minimal’ from the text, see line 158. Although EdU labelling does not provide a direct measurement of proliferation rates, our results may implicate temporally faster proliferation of BECs than hepatocytes at 96 and 120 hpf. On the other hand, cell counts, an alternative and robust method to determine cell number dynamics, show that *in vivo* the proportion of BECs at 96 and 120 hpf does not increase relative to the time points before and after (Fig. 2G), suggesting a balancing mechanism. Nevertheless, testing the effect of a transient difference in proliferation on the total cell type proportions, we modified model 6, which displays proportions closest to the ones observed *in vivo*. More specifically, starting from the time point when the simulations reached 2300 cells (corresponding to 96 hpf *in vivo*) we reduced BEC doubling time by 2.9-fold (corresponding to an average of 2.23-fold at 96 hpf, and 3.58-fold at 96 hpf). This results in a BEC to hepatocyte ratio of 1:5.2 at the end of the modelling period, when 3000 cells correspond to about 120 hpf (see model below). As expected, the shorter BEC cell doubling time takes the cell type proportion in the model away from the proportions observed *in vivo*.

In summary, integrating temporally increased BEC proliferation in mathematical model 6 does not alter the initial finding that cell type proportions (Fig. 2G) are set up early by lineage decisions, yet it provides first evidence for a mechanism that responds to temporal variances in cell type proportions to sustain liver tissue organisation.

This is now mentioned in the text and discussion (lines 152-154 and 417-420).

2)      In response to query F: The explanation provided in the rebuttal regarding the temporal differences in clone detection due to differences in maturation of the distinct fluorescent cassettes used should be stated in the MS. This allows the reader to accurately understand the dynamics of the labeling system.

We agree with the reviewer. To keep the manuscript easy to follow, yet provide this important technical information, we have added the information to the legend of the figure showing the corresponding data (Figure S2).

*“Temporal FP colour detection reflects the individual protein maturation times and depends on the strength of the respective Gal4-driver [32].”*

3)      In response to question posed in query F (and in query J): "Could the higher than expected number of small clones reflect a later than expected onset of recombination?"  This question has not been answered. I am not referring to the number of clones observed in non-heat shocked control animals (which are referred inaccurately in the MS as "unspecific recombination events", unspecific is not an appropriate word), although this is an essential parameter that should have originally been included in the MS.  What are the data supporting the stated maturation of PhiC31 for the 4 hours after heat shock (26-30 hpf) and the attB/P recombination confined to the 3 hours after that (30-33 hpf). Where are the experimental details that support this narrow time-frame? Is it possible that recombination occurs later than this because of delayed PhiC31 maturation or because of a longer than expected life of the attB/P recombinase?

We apologise for not making it clear in the first revision, below we try to clarify and address it in a detailed reply:

a. The word ‘unspecific’ was replaced throughout the manuscript with ‘non-specific’; lines 211, 258, 310)
b. The FRaeppli recombination timeline of 6-7 hours is supported by following data:

PhiC31 maturation of 4 hours is described in the original publication describing the FRaeppli system and referenced in line 196 (reference #32). Specifically, by investigating mRNA and protein expression dynamics using mRNA in situ hybridization and *hsp70l:phiC31-sfGFP-NLS*, respectively, we had demonstrated expression of *phiC31*mRNA between 2 and 4 hrs and of PhiC31 protein 4 hrs post heat-shock.

Furthermore, based on the valid points raised in the previous rebuttal, we had designed PCR experiments to monitor the temporal dynamics of *fraeppli-nls* recombination in embryos, by assessing recombined genomic loci of the individual fluorescent proteins at multiple timepoints after heat-shock-mediated induction of phiC31 expression. Following heat-shock-mediated induction of phiC31 expression at 26 hpf, all embryos exhibited at 33 hpf, seven hours after phiC31 induction, successful recombination and most embryos of all four fluorescent proteins. These data had been added as panels E and F to Fig. S2. These temporal dynamics of recombination mediated by exogenous phiC31 are similar to those in the early embryo (Caviglia et al. 2022) and thus likely a general characteristic of the system.

In summary, these data provided the basis for the initially indicated 6-7 hrs period between heat-shock and FRaeppli recombination.

c. However, considering the point of PhiC31 protein stability, PhiC31-sfGFP-NLS is detectable after its first appearance and therefore later FRaeppli cassette recombination cannot be excluded. For this reason, we had included the statement that later recombination events are formally possible. We have now added this detail to make it more explicit to the statement, which now *reads: “An overrepresentation of small clones caused by later than expected labelling events due to lingering PhiC31 protein represents a formal possibility, however, is less likely given that PhiC31 recombination occurs quickly and efficiently (Figs. S2E,F). ~~and low non-specific recombination (1.8%) observed in controls~~.“* See text lines: 259. In addition, we have replaced the continuous lines initially marking the time periods in Figure S2D with dotted lines to indicate that protein maturation and recombination could occur beyond.
d. Moreover, information on the non-specific recombination events had already been part of the initial manuscript, preceding the description of the clonal analysis (page 8; now: page 9 line 210), and we acknowledge that it indeed is not part of this argument.

4)      In response to query F and J: The revised text inserted in lines 250-253 is not stated in a clear manner (and in response to the new comment 3 may still need to be updated). "An overrepresentation of small clones caused by later than expected labelling events represents a formal possibility, however, less likely given that PhiC31 recombination occurs fast and efficient (Figs S2E,F) and low unspecific recombination (1.8%) observed in controls." Instead of "Recombination occurs fast and efficient" perhaps recombination occurs quickly and efficiently". Furthermore, "Unspecific" is not an appropriate word to use (perhaps you mean non-specific?).

Following the reviewers suggestion, we have modified the sentence (modifications are indicated by underlining), which now reads (lines 256-258): *"An overrepresentation of small clones caused by later than expected labelling events due to lingering PhiC31 protein represents a formal possibility, however, is less likely given that PhiC31 recombination occurs quickly and efficiently (Figs S2E,F) ~~and low non-specific recombination (1.8%) observed in controls~~."*

5)      In response to query G: In Figure S2 it appears as if exogenous PhiC31 mRNA injection is used in H and I. However you state in your rebuttal that only exogenous phiC31 under the control of hsp70l promoter is used. Please ensure that the details for such exogenous mRNA injection are included in the methods and are overtly stated in the legend for Fig S2.

Thank you for pointing this out, we missed this detail, which is now stated in the corresponding methods section (lines 645-647) and in the legends accompanying Figure S2 (line 38).

6)      The revisions provided in the new text on lines 179-199 needs to be better edited for accuracy.

Line 180 "The spectra…" I think that a more accurate way of stating what you mean is: " Thes spectra of FRaeppli FPs does not overlap with the spectra of EGFP allowing for the simulataneous use of the transgenic tp1:EGFP…"

We have changed the text as follows:

*“The spectra of the FRaeppli FPs are distinct from the EGFP spectra allowing for the simultaneous use with transgenic tp1:EGFP expressed in BECs.“*

Line 181-183 "To ensure… we used prox1 as a driver" insert "as a UAS driver”

Integrating the idea of the reviewer’s suggestion, we provided more information on the type of driver line. In this case it is a KalTA4-driver and the UAS-lines are responders.

*“To ensure that the UAS:fraeppli-nls transgene (short: fraeppli-nls) is expressed in progenitors and maintained in hepatocytes and BECs, we used prox1a:kalTA4 as a KalTA4-driver (Figs. 3A, S3A), since Prox1 is expressed in hepatoblasts and differentiated hepatocytes and BECs (Fig.S2A-C) [33]. “*

Line 186-188 "For conditional induction of recombination…for conditional induction of cell recombination".  Please make this sentence more accurate and concise.

*“Conditional recombination of the FRaeppli-cassette in individual cells prior to cell differentiation in fraeppli-nls was achieved with hsp70l:phiC31 and induced by heat-shock at 26 hpf (Figs. 3B-C).”*

Lines 188-191: As per the timing question posed in Query 4 above, the sentence beginning with "PhiC21 maturation and subsequent attB/P recombination….takes about 6-7hours (note: insert space between 7 and hours) and thus occurs prior to fate commitment" unless evidence excludes later recombination occurring as a result of PhiC21 the statement should be "thus initiates prior to fate commitment”.

The sentence has been updated to:

*“PhiC31 maturation and subsequent attB/attP recombination, initiating the stable expression of one of four fluorescent proteins per cell, takes about 6-7 hours (Figs. 3B-C, S2D-F) [32] and thus initiates prior to fate commitment.”*
